# The impact of using BARCIST 1.0 criteria on quantification of BAT volume and activity in three independent cohorts of adults

**DOI:** 10.1038/s41598-018-26878-4

**Published:** 2018-06-04

**Authors:** Borja Martinez-Tellez, Kimberly J. Nahon, Guillermo Sanchez-Delgado, Gustavo Abreu-Vieira, Jose M. Llamas-Elvira, Floris H. P. van Velden, Lenka M. Pereira Arias-Bouda, Patrick C. N. Rensen, Mariëtte R. Boon, Jonatan R. Ruiz

**Affiliations:** 1 PROFITH “PROmoting FITness and Health through physical activity” research Group, Department of Physical Education and Sport, Faculty of Sport Sciences, University of Granada, Granada, Spain; 2 Department of Medicine, Division of Endocrinology, and Einthoven Laboratory for Experimental Vascular Medicine, Leiden University Medical Center, Leiden, The Netherlands; 3 Servicio de Medicina Nuclear, Hospital Universitario Virgen de las Nieves, Granada, Spain; 4 Instituto de Investigación Biosanitaria (ibs. GRANADA), Servicio de Medicina Nuclear, Granada, Spain; 5 Department of Radiology (Division of Nuclear Medicine), Leiden University Medical Center, Leiden, The Netherlands; 6 Department of Nuclear Medicine, Alrijne hospital, Leiderdorp, The Netherlands

## Abstract

Human brown adipose tissue (BAT) is commonly assessed by cold-induced ^18^F-fluorodeoxyglucose (FDG) PET-CT using several quantification criteria. Uniform criteria for data analysis became available recently (BARCIST 1.0). We compared BAT volume and activity following BARCIST 1.0 criteria against the most commonly used criteria [Hounsfield Units (HU):-250, -50, standardized uptake value (SUV):2.0; HU: Not applied, SUV:2.0 and HU:-180, -10, SUV:1.5] in a prospective study using three independent cohorts of men including young lean adults, young overweight/obese adults and middle-aged overweight/obese adults. BAT volume was the most variable outcome between criteria. While BAT volume calculated using the HU: NA; SUV: 2.0 criteria was up to 207% higher than the BAT volume calculated based on BARCIST 1.0 criteria, it was up to 57% lower using the HU: -250, -50; SUV: 2.0 criteria compared to the BARCIST 1.0. Similarly, BAT activity (expressed as SUV_mean_) also differed between different thresholds mainly because SUV_mean_ depends on BAT volume. SUV_peak_ was the most consistent BAT outcome across the four study criteria. Of note, we replicated these findings in three independent cohorts. In conclusion, BAT volume and activity as determined by ^18^F-FDG-PET/CT highly depend on the quantification criteria used. Future human BAT studies should conduct sensitivity analysis with different thresholds in order to understand whether results are driven by the selected HU and/or SUV thresholds. The design of the present study precludes providing any conclusive threshold, but before more definitive thresholds for HU and SUV are available, we support the use of BARCIST 1.0 criteria to facilitate interpretation of BAT characteristics between research groups.

## Introduction

Brown adipose tissue (BAT) is present and metabolically active in human adults^1^. In 2009, several studies using ^18^F-fluorodeoxyglucose (FDG) positron emission tomography (PET) combined with X-ray computed tomography (CT) (^18^F-FDG-PET/CT) imaging showed that human BAT can be activated upon cold exposure, that ^18^F-FDG uptake by BAT is more common in women than in men, and that ^18^F-FDG uptake by BAT is higher in lean than in obese individuals^2–5^. The capacity of BAT to combust energy as well as its beneficial role in glucose^6^ and lipid^7^ metabolism makes BAT an attractive therapeutic target in combating adiposity and type 2 diabetes.

Currently, ^18^F-FDG-PET/CT analysis is the most commonly used method to quantify human BAT volume and activity^1^. ^18^F-FDG-PET provides information about glucose uptake by metabolically active tissues including BAT, expressed as standardized uptake value (SUV)^8,9^. CT, on the other hand, provides anatomical information and allows the identification of various tissues, including adipose tissue and soft tissues, based on the radio-density expressed as Hounsfield units (HU). Therefore, quantification of BAT volume and activity depends to a great extent on the selection and combination of HU and SUV thresholds^1^. Due to the lack of consensus on the most appropriate HU and SUV thresholds to quantify BAT volume and activity in humans, studies have shown different levels of BAT volume and activity in cohorts with similar characteristics^10,11^. Consequently, since optimal thresholds are not known, current available data on human BAT volume and activity are speculative at best. Moreover, the use of different HU and SUV thresholds hampers comparability across studies.

Recently, an expert panel launched a set of recommendations for conducting ^18^F-FDG-PET/CT analysis of human BAT (Brown Adipose Reporting Criteria in Imaging Studies, BARCIST 1.0)^1^. BARCIST 1.0 recommends using a HU range between -190 and -10, and a SUV threshold of [1.2/(lean body mass (LBM)/body mass (BM))]. The impact of using the BARCIST 1.0 HU and SUV thresholds compared to the most commonly used protocols to quantify BAT volume and activity is currently unknown since, to our knowledge, no studies using these specific thresholds have been reported yet. It is of interest to better understand the relative differences obtained with BARCIST 1.0 thresholds compared to the currently most commonly used thresholds in literature for populations with different age and BMI.

Therefore, in the present study we aimed to compare and quantify BAT volume and activity following BARCIST 1.0 recommendations against the most commonly used HU and SUV thresholds in three different cohorts of men including young lean adults, young overweight/obese adults, and middle-aged overweight/obese adults.

## Methods

### Participants

A total of thirty men from three independent cohorts were included in this prospectively designed study. Participants’ characteristics are shown in Table 1. The study cohorts were: (i) 10 young lean adults (21–29 years old; BMI 19–24 kg/m^2^, white Caucasians)^12^, (ii) 10 young overweight/obese adults (18–25 years old; BMI 25–35 kg/m^2^)^13^, and (iii) 10 middle-aged overweight/obese adults (35–53 years old; BMI 26–30 kg/m^2^). The study conducted in young overweight/obese adults was approved by the Human Research Ethics Committee of both University of Granada (n°924) and Servicio Andaluz de Salud (Centro de Granada, CEI-Granada). The Medical Ethical Committee of the Leiden University Medical Center approved the other study cohorts (2473 and NCT02294084). All volunteers provided written informed consent before participation. All studies were performed in accordance with the Declaration of Helsinki.

**Table 1:** Characteristics of participants and study conditions of the three cohorts.

	Young lean adults (N = 10)	Young overweight/obese adults (N = 10)	Middle-aged overweight/obese adults (N = 10)
Age (years)	25 ± 3	22 ± 2	42 ± 6
Height (m)	1.85 ± 0.05	1.78 ± 0.09	1.80 ± 0.05
Weight (kg)	76.0 ± 7.3	92.9 ± 16.1	89.9 ± 7.6
BMI (kg/m^2^)	22.2 ± 1.6	29.0 ± 3.4	27.8 ± 1.3
Waist-to-hip ratio	0.87 ± 0.04	0.90 ± 0.08	0.96 ± 0.04
Fat mass (kg)	13.4 ± 4.2	31.7 ± 9.7	24.7 ± 6.5
Lean body mass (kg)	59.6 ± 5.9	56.2 ± 7.3	61.9 ± 5.1
Fasting glucose (mmol/l)	4.2 ± 0.3	5.0 ± 0.4	4.7 ± 0.4
Study period	March 2013 to June 2013	October 2015 to November 2016	March 2015 to June 2016
Country	The Netherlands	Spain	The Netherlands
Cooling methodology	2 water-perfused blankets (Blanketrol III, Cincinnati USA)	1 water-cooling vest (Polar Products Inc., Ohio, USA) with light cool air conditioned room.	2 water-perfused blankets (Blanketrol III, Cincinnati USA)
Time of cold exposure prior PET/CT scan	Shivering test + 2 h to individualized mild cold	Shivering test (48–72 h before) + 2 h to individualized mild cold	Shivering test + 2 h to individualized mild cold
PET/CT scanner	Gemini TF64 PET/CT, Philips, Netherlands	Siemens Biograph 16 PET/CT, Siemens, Germany	Gemini TF64 PET/CT, Philips, Netherlands
DEXA scan	Lunar iDXA, GE Healthcare, UK	QDR 4500 W, HOLOGIC, USA	Lunar iDXA, GE Healthcare, UK
^18^F-FDG (MBq)	152 ± 15	188 ± 11	107 ± 4
Ratio ^18^F-FDG (MBq) to BMI (kg/m^2^)	6.8 ± 0.3	6.5 ± 0.8	3.8 ± 0.3
SUV lean body mass ^1^ threshold (g/ml)	1.54 ± 0.09	1.98 ± 0.17	1.75 ± 0.15
SUV_mean_ (g/ml) of descending aorta (reference tissue)	1.18 ± 0.17	1.62 ± 0.23	1.66 ± 0.29

Data are means ± standard deviation.

BMI: Body mass index; DEXA: Dual Energy X-ray Absorptiometry; ^18^F-FDG: ^18^F-fluorodeoxyglucose; MBq: Megabecquerel; PET/CT: Positron Emission Tomography/Computed Tomography; SUV: Standardized uptake value.

### Systematic review on most commonly used thresholds

To identify the most commonly used HU and SUV thresholds for ^18^F-FDG-PET/CT scans in BAT research, we conducted a systematic literature search on MEDLINE (from January 1^st^ 2007 to March 10^th^ 2017) for studies reporting human BAT volume and activity. We used the Medical Subject Heading (MeSH) terms “Adipose Tissue, Brown” in combination with type of population (men, women, and adults) and instrument used (“PET/CT”, “PET-CT”, and “18-FDG”). We excluded human studies that did not use ^18^F-FDG-PET/CT scans to assess BAT volume and activity, studies conducted in animal models or not written in English, and reviews. In addition, we searched the reference lists of all identified relevant publications. No restrictions were considered regarding study design (cross sectional, case-control, cohort study) or data collection (prospective or retrospective). To avoid duplicate data, we identified articles that included the same group of participants by reviewing inter-study similarities in any of the following characteristics: country in which the study was conducted, investigators who performed the study, source of patients, recruitment period, and inclusion criteria. When the same investigators reported results obtained on the same group of patients in several publications, only the first published study was included.

### Cooling protocol

To activate BAT, we applied personalized cooling protocols prior to ^18^F-FDG-PET/CT scans. Slightly different water-cooling methods were used between the different cohorts due to differences in local equipment and protocols. In the young lean and middle-aged overweight/obese cohorts two water-perfused temperature-controlled mattresses (Blanketrol III, Cincinnati Sub-Zero Products, Cincinnati, OH, USA) were used, as previously described^12^. In short, participants were sandwiched between two water-perfused mattresses starting at a temperature of 32 °C, which was subsequently gradually decreased. When shivering occurred (after 30–40 min), temperature was raised by 3–4 °C and a stable cooling period of 2 h started. In the cohort of young overweight/obese adults a water perfused vest (Polar Products Inc., Ohio, USA) was used^14^. Shivering threshold was determined 48–72 h before the ^18^F-FDG-PET/CT scan. Immediately before the ^18^F-FDG-PET/CT scan, participants wore the water perfused vest for 2 h set at approx. 4 °C above the temperature that caused the onset of shivering. In all three studies, after 1 h of stable cooling ^18^F-FDG was injected (Table 1) and after 2 h of cooling ^18^F-FDG-PET/CT imaging was performed.

### Body composition

Fat mass and lean body mass were measured by Dual Energy X-ray Absorptiometry (DEXA) in the three cohorts (Table 1).

### ^18^F-FDG-PET/CT scan

Cold-induced BAT volume and activity were assessed by ^18^F-FDG-PET/CT scanning. Table 1 shows details of the PET/CT scanners as well as doses of ^18^F-FDG injected in each study cohort. In the three studies, for the CT acquisition a peak kilovoltage of 120 was applied, while for the PET acquisition a scan time of 6 min per bed position was set. In total, 6 bed positions were scanned for the young lean and middle-aged overweight/obese adults (from top of the head to pelvis) and 2 bed positions for the young overweight/obese adults (from *atlas vertebra* to mid-chest, which was a requirement set by the Human Research Ethics Committee to limit radiation burden).

### Quantification of human BAT volume and activity

PET/CT images were analyzed using the Beth Israel plugin for FIJI^2^ software by two trained researchers (BMT and KJN)^15^. Disagreements between both researchers were arranged in a consensus meeting. The regions of interest (ROIs) were semi-automatically outlined from *atlas vertebrae* (Cervical 1) to *thoracic vertebrae* 4 (see Figure S1) using a 3D-Axial technique^11^. The ROIs were placed in this range because (i) this region predominantly shows BAT activity and (ii) it is the region that is consistently scanned in all three study cohorts. Regions such as mouth, nose, or thyroid were not included to avoid potential false positives within the ROIs. We calculated the standardized uptake value (SUV) as [^18^F]-FDG uptake (kBq/mL)/(injected dose [kBq]/patient weight [g])]. We defined BAT volume, SUV_mean_, BAT metabolic activity, SUV_max_ and SUV_peak_ following BARCIST 1.0 criteria^1^. In short, BAT volume was calculated as the sum of the volumes identified as BAT in each ROI (1 to 6, see Figure S1). SUV_mean_ was calculated by the weighted average of SUV_mean_ derived from each ROI (1 to 6). SUV_peak_ was the highest average SUV in a 1 ml spherical volume. This sphere may, or may not, be centered on the highest SUV_max_ over all ROIs (1 to 6). We also drew a ROI on the descending aorta as reference tissue (Table 1)^1^. To quantify BAT, we applied the criteria of BARCIST 1.0, in addition to the three most used combinations of thresholds detected in the systematic review (see below).

### Statistical analysis

Differences across HU and SUV thresholds were analyzed using analysis of variance (ANOVA) with Bonferroni adjustments for post-hoc comparisons. Separate analyses were conducted for each study cohort. The inter-observer (BMT and KJN) reliability was assessed using Lin’s concordance coefficient (LCC)^16^. All analyses were conducted using the Statistical Package for Social Sciences (SPSS, v. 22.0, IBM SPSS Statistics, IBM Corporation) and the level of significance was set to ≤0.05. Data are presented as mean ± standard deviation unless otherwise stated.

## Results

### Characteristics of participants

Table 1 summarizes the characteristics of the participants and the study conditions in the three cohorts.

### Systematic review: Selection of HU and SUV thresholds

After having read titles and abstracts, 344 studies were excluded from a total of 471; 131 were checked for relevance (full text); and 123 met the inclusion criteria. After excluding redundant studies, i.e. studies that used the same participants and images, a total of 116 studies were finally included (Table S1). Table 2 shows a summary of the HU and SUV thresholds used in the quantification of human BAT by ^18^F-FDG-PET/CT. We found nine different combinations of HU and SUV thresholds published twice or more, and 26 different combinations of HU and SUV thresholds published just once. HU and/or SUV thresholds were not mentioned in as many as 32 studies. We selected the three most frequently used combinations of HU and SUV thresholds (Table 2): HU: -250, -50; SUV: 2.0, HU: N.A.; SUV: 2.0 and HU: -180, -10; SUV: 1.5 for comparison with the BARCIST 1.0 criteria.

**Table 2:** Summary of Hounsfield units (HU) and standardized uptake value (SUV) thresholds used for quantification of human brown adipose tissue by ^18^F-FDG-PET/CT scans from January 1, 2007 to March 10, 2017.

HU	SUV ≥	No. of studies: 116
-250; -50	2.0	20
Not used/Not reported	2.0	10
-180; -10	1.5	7
-300; -10	2.0	4
-100; -10	1.0	3
-250; -10	2.0	3
-150; -30	1.5	2
-250; -50	SUV different at 2.0	7
-180; -10	SUV different at 1.5	2
Other combinations		26
Not reported	Not reported	32

See Table S1 for references and details of Hounsfield units, standardized uptake value, and software used to quantify human brown adipose tissue.

### High inter-observer reliability was found regardless of thresholds applied

Lin’s concordance coefficient (LCC) of BAT volume and in the three cohorts was above 0.950, except in one case (0.906; SUV_peak_ in overweight/obese adults; HU: NA, SUV: 2.0; Table S2). When BARCIST 1.0 criteria were applied, the LCC were 0.962–0.996, 0.980–0.996 and 0.983–1.000, for BAT volume, SUV_mean,_ and SUV_peak_, respectively (Table S2).

### The combination of HU and SUV thresholds markedly affects estimation of BAT volume and activity across different cohorts

**Figure 1:**
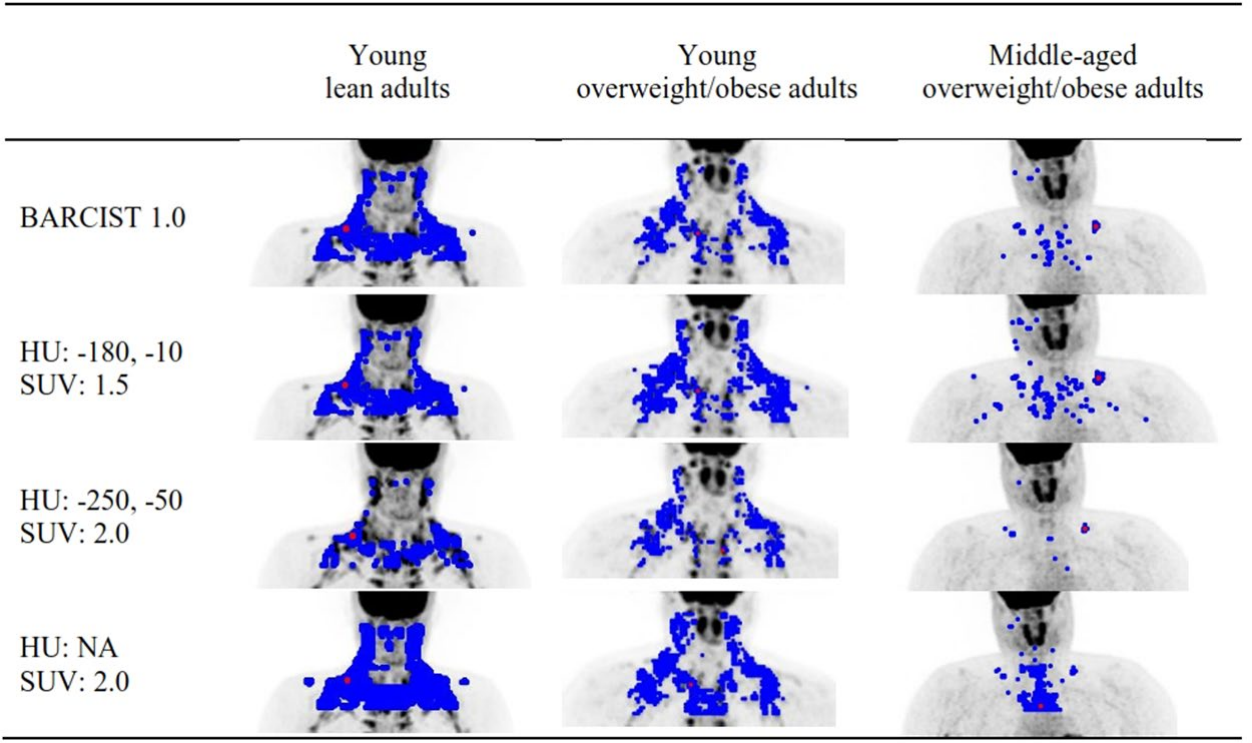
Representative images of brown adipose tissue volume and activity of the three study cohorts by threshold of Hounsfield units (HU) and standardized uptake value (SUV). Blue dots indicate BAT volume and red dots indicate maximal BAT activity (SUV max). BMI: Body mass index; HU: Hounsfield units; NA: Not applied; SUV: Standardized uptake value.

**Figure 2:**
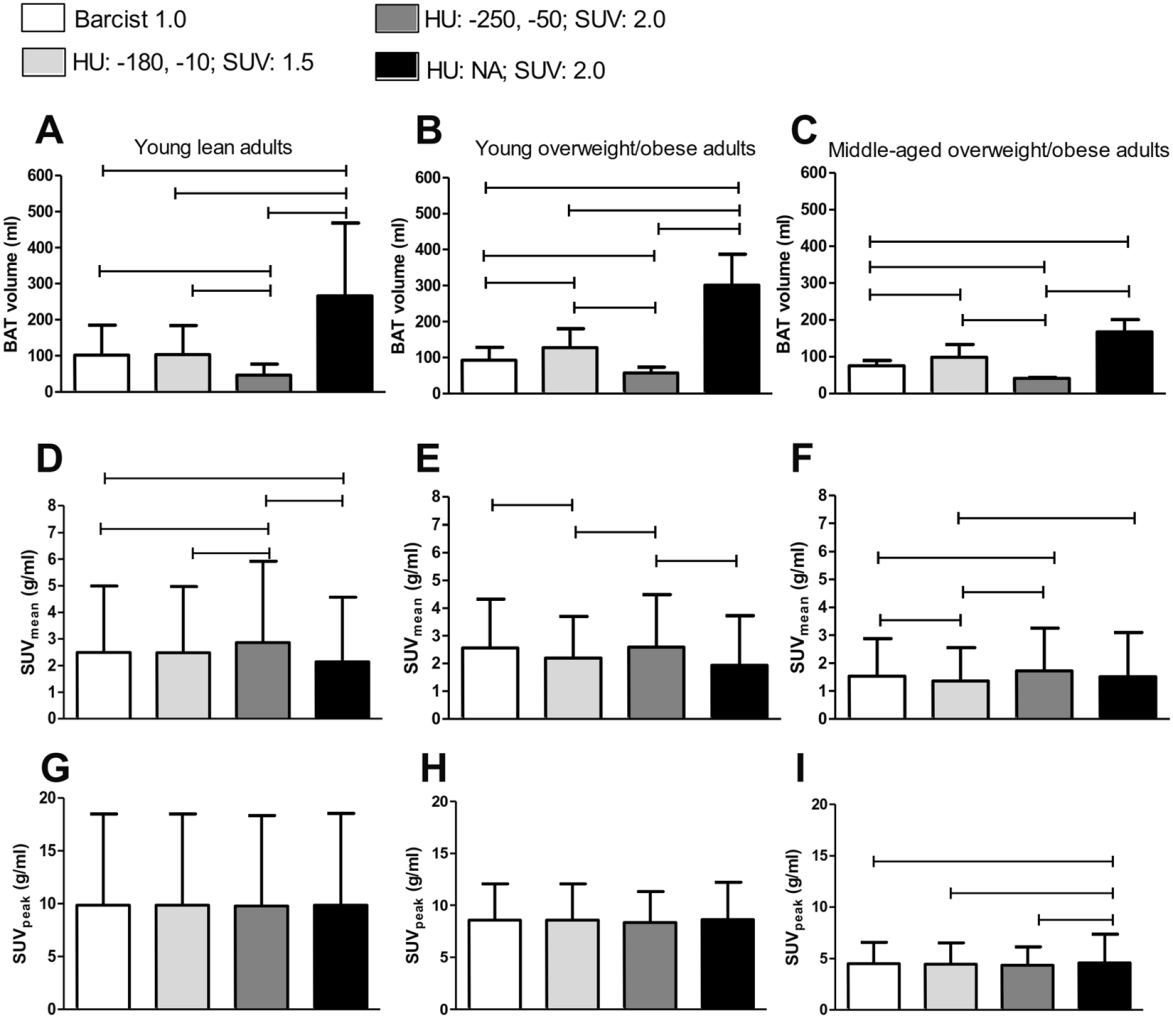
Brown adipose tissue (BAT) volume and activity determined by various thresholds of Hounsfield unit (HU) and Standardized uptake value (SUV) for three study cohorts. BAT volume (**A**–**C**), SUV_mean_ (**D**–**F**), and SUV_peak_ (**G**–**I**) were determined in young lean adults (**A**,**D**,**G**), young overweight/obese adults (**B**,**E**,**H**), and middle-aged overweight/obese adults (**C**,**F**,**I**). Data are means and standard deviation (n = 10 per cohort). Significant differences between thresholds are indicated by parallel horizontal bars (all P ≤ 0.05). BARCIST 1.0: HU:-190, -10; SUV: Individualized [1.2/(lean body mass/body mass)]; BMI: Body mass index; NA: Not applied. See Table S4 for exact absolute and relative differences between thresholds.

Representative images of BAT volume and activity of the three study cohorts resulting from different HU and SUV threshold combinations are shown in Fig. 1. Firstly, we determined the quantitative effect of the four threshold combinations on BAT volume in young lean adults (Fig. 2A), young overweight/obese adults (Fig. 2B), and middle-aged overweight/obese adults (Fig. 2C). Compared to the BARCIST 1.0 criteria, higher BAT volumes were consistently observed using the HU: NA; SUV: 2.0 criteria in young lean adults [+155%; 249 (186, 312) ml; mean (95% CI)], young overweight/obese adults [+207%; 244 (114, 374) ml], and middle-aged overweight/obese adults [+124%; 106 (42, 170) ml]. In contrast, BAT volumes estimated with the HU: -250, -50; SUV: 2.0 criteria compared to the BARCIST 1.0 criteria, yielded significantly smaller values in young lean adults [-57%; 92 (79, 106) ml], young overweight/obese adults [-42%; 49 (29, 70) ml], and middle-aged overweight/obese adults [-54%; 46 (28, 64) ml] (see Table S3). No significant differences were observed between BAT volumes estimated by HU: -180, -10; SUV: 1.5 criteria compared to BARCIST 1.0 criteria in young lean adults [0%; 0 (-5, 5) ml], however, we found higher BAT volumes in young overweight/obese adults [+40%; 47 (24, 69) ml] and middle-aged overweight/obese adults [+45%; 38 (18, 58)].

### HU and SUV thresholds moderately affect estimation of BAT activity (SUV_mean_) across different cohorts

We next determined the effect of the four threshold combinations on SUV_mean_ in young lean adults (Fig. 2D), young overweight/obese adults (Fig. 2E), and middle-aged overweight/obese adults (Fig. 2F). Compared to the BARCIST 1.0 criteria, higher SUV_mean_ were observed using the HU: -250, -50; SUV: 2.0 criteria in young lean adults [+18%; 0.8 (0.9–0.6) g/ml] and in middle-aged overweight/obese adults [+13%; 0.3 (0.2–0.5) g/ml]. In young lean adults, a lower SUV_mean_ was estimated using the HU: NA; SUV: 2.0 criteria compared to BARCIST 1.0 criteria [-9%; 0.4 (0.2, 0.6) g/ml]. Lower BAT activity was detected using HU: -180, -10; SUV: 1.5 criteria against BARCIST 1.0 criteria in young overweight/obese adults [-14%; 0.5 (0.4, 0.7) g/ml] and middle-aged overweight/obese adults [-11%; 0.3 (0.2–0.4) g/ml] (Table S3).

### HU and SUV thresholds slightly affect estimation of maximal BAT activity (SUV_peak_) values across different cohorts

In addition, we determined the effect of the four threshold combinations on SUV_peak_ in young lean adults (Fig. 2G), young overweight/obese adults (Fig. 2H), and middle-aged overweight/obese adults (Fig. 2I). We found similar results in the SUV_peak_ values (all P > 0.05), however, there were significant differences in the middle-aged overweight/obese adults, where SUV_peak_ values calculated with HU: NA; SUV: 2.0 criteria were higher than calculated with BARCIST 1.0 criteria [+10%; 0.6 (0.2, 0.9) g/ml]. Figure S2 shows means and 95% CI of BAT volume and activity determined by various thresholds of HU and SUV for the three study cohorts.

## Discussion

To our knowledge, the present study is the first empirical investigation to compare and quantify BAT volume and activity following BARCIST 1.0 criteria against the most used HU and SUV thresholds in three independent cohorts of men of different age and BMI. We observed that BAT volume calculated using the HU: NA; SUV: 2.0 criteria may differ up to 2.6-fold, 3.1-fold, and 2.2-fold from the BAT volume calculated based on BARCIST 1.0 criteria in young normal-weight, young overweight/obese, and middle-aged overweight/obese adults, respectively. Similarly, BAT activity (expressed as SUV_mean_), based on BARCIST 1.0 criteria differed up to 18% and 13% when compared with BAT activity based on the HU: -250, -50; SUV: 2.0 criteria, for young lean adults and middle-aged overweight/obese adults, respectively. On the other hand, differences were not significant when maximal BAT activity was expressed as SUV_peak_ for young cohorts, while we found that SUV_peak_ based on the HU: NA; SUV: 2.0 was 10% higher than calculated with BARCIST 1.0 criteria in middle-aged overweight/obese adults. The findings of this study show that human BAT volume and to a lesser extent BAT activity (expressed as SUV_mean_) depend largely on the selected HU and SUV thresholds. Moreover, we observed that the various threshold combinations similarly influenced BAT volume and BAT activity (SUV_mean_) in the three cohorts independently of age, BMI or different methodologies applied to activate and measure BAT^17^.

### HU and SUV thresholds markedly affect BAT volume across different cohorts

The scientific community has been speculating about the volume of BAT that is present in adult men, and showed mean values of approx. 70 ml^3^, 100 ml^2,18^, 300 ml^19^, and 450 ml^20^. This highlights the knowledge gap regarding the amount of BAT present in adults and that maximizing its volume and metabolic activity could impact human physiology. We found that the highest BAT volumes were consistently observed with those thresholds that did not apply a criterion for HU (i.e. HU: NA, SUV: 2.0). Because the gold standard to quantify BAT is the ^18^F-FDG-PET in combination with CT, it is evident that a threshold of HU should be applied, although the most appropriate threshold of HU remains to be elucidated. We observed lower BAT volumes with the most frequently used threshold combination in literature (HU: -250, -50, SUV: 2.0) compared to BARCIST 1.0. These differences could be based on the fact that BARCIST 1.0 uses an HU range starting at -10 while the most commonly protocol used an HU range starting at -50, albeit it is known that the range of adipose tissue in the CT images starts at an HU value of -10^11,21^. Moreover, it was recently shown^22^ that the density of the BAT might change after a cold exposure, especially in the range from -50 to -10. Actually, it is not feasible to distinguish BAT from WAT or other tissues using exclusively CT criteria. Thus, more studies are needed to increase our understanding of BAT density measured by a CT scan^23^.

The main advantage of the BARCIST 1.0 recommendations is the inclusion of an individualized SUV threshold adapted to the individual’s lean body mass. Estimated BAT volumes did not differ between BARCIST 1.0 and HU: -180, -10; SUV: 1.5 criteria in the young lean adults, probably because the SUV criteria were virtually identical using both thresholds (1.54 ± 0.09 vs. 1.5 g/ml, respectively). Nevertheless, we found higher estimated BAT volume with HU: -180, -10; SUV: 1.5 compared to BARCIST 1.0, because the SUV threshold applied in BARCIST was higher for young overweight/obese adults and middle-aged overweight/obese adults (1.98 ± 0.17 g/ml and 1.75 ± 0.15 g/ml, Table 1). Therefore, the relative changes in BAT volume are largely influenced by the use of a SUV threshold corrected by lean body mass^1,11^.

### HU and SUV thresholds moderately affect BAT activity (SUV_mean_) across different cohorts

SUV_mean_ is the average SUV within a volume, therefore, if BAT volume differs due to HU or SUV thresholds in the three cohorts, SUV_mean_ is also expected to differ by the thresholds selection. Indeed, SUV_mean_ is not consistent across studies as it is influenced by many methodological factors as described previously^17^ such as cooling protocols or instruments, as well as study populations^8,9^. We showed that when a SUV individualized threshold was used and the SUV values are alike (such as HU: -180, -10; SUV: 1.5 criteria in young lean adults), then the SUV_mean_ values are similar. However, we observed that an application of a higher SUV threshold results in higher SUV_mean_ values, regardless of the HU thresholds applied or the cohort studied because the SUV threshold of 2.0 includes higher ^18^F-FDG uptake of BAT deposits. On the other hand, when an HU threshold was omitted (i.e. HU: NA; SUV: 2.0 criteria), the SUV_mean_ values were lower regardless of the cohort studied. In this case, all ^18^F-FDG present in the selected ROIs of the PET images is used for quantification of BAT activity even though part of ^18^F-FDG would represent uptake by other tissues that were not excluded for the analysis due to lack of HU criteria.

### HU and SUV thresholds slightly affect maximal BAT activity (SUV_peak_) values across different cohorts

BARCIST 1.0 recommends to report SUV_peak_ instead of SUV_max_ to avoid overestimation in the quantification of BAT activity^1^, because SUV_max_ is the single highest uptake pixel in the ROI and could easily be an outlier whereas SUV_peak_ is the highest average SUV in a 1 cc spherical volume, thereby reducing a potential effect of outliers. This sphere may, or may not, be centered on the highest SUV_max_. Letiner *et al*.^11^ found that PET image resolution substantially influences observed BAT SUV_max_ but whether this resolution also affects SUV_peak_ in currently unknown. Compared to the BARCIST 1.0 criteria, we found similar SUV_peak_ values across thresholds in young lean men and overweight/obese men. However, differences were observed between the thresholds that did not use HU vs. all the other thresholds in middle-aged overweight-obese adults. This could be based on the lower amount of ^18^F-FDG injected with respect to the size/body weight of the participant [ratio between amount of ^18^F-FDG to BMI (6.8 ± 0.3, 6.5 ± 0.8, and 3.8 ± 0.3 MBq/(kg/m^2^) in young lean adults, young overweight/obese adults, and middle-aged overweight/obese adults, respectively]. Therefore, the distribution of the tracer among the various tissues may partially explain this finding. In fact, lower doses of ^18^F-FDG, as used in the cohort of middle-aged overweight/obese adults, may increase noise in the image and, therefore, raise SUV_peak_ levels^1,8,9^. We found that SUV_max_ was located in BAT regions irrespective of the threshold used in young lean and young overweight/obese men. However, in middle-aged overweight/obese men, SUV_max_ was found in an unexpected region when no threshold for HU was used. Therefore, omission of HU threshold may have resulted in an artificial SUV_max_ and consequently SUV_peak_, especially in the middle-aged overweight-obese men who received a low dose of ^18^F-FDG. Similar differences between thresholds and cohort studies were found with SUV_max_ (data not shown). Therefore, in light of these findings, we support that SUV_peak_ is the most consistent BAT-related outcome between criteria in the three independently cohorts of adults.

### Limitations

We quantified BAT in six different ROIs from *cerebellum* to *thoracic vertebra 4* (Figure S1). Although most of the BAT detected in humans is localized in the areas covered by the selected ROIs^11^, we may have missed BAT depots in axillary, paraspinal or abdominal adipose tissue located in anatomical areas beneath the *thoracic vertebra 4*^11^. Our ROIs did not include mouth, nose, or thyroid to avoid false positive results, yet, results persisted when a single ROI from *cerebellum* to *thoracic vertebrae 4* was drawn and when HU criteria were applied (data not shown). In addition, we do not know if these findings can be replicated when the SUV threshold of BARCIST criteria is used in combination with other ranges of HU. Besides the selection of HU and SUV thresholds, quantification of human BAT volume and activity also depends on other methodological issues such as the cooling protocol, ^18^F-FDG-PET/CT methodology, segmentation software^17^, tracer used (^18^F-FDG vs. ^18^F-FTHA^24^), intrinsic factors of the participants such as age, sex, or body composition, or extrinsic factors as outdoor temperature^25^ or daily light^26^, which limit comparisons across studies. To improve the understanding of human BAT measured by ^18^F-FDG-PET/CT, the reconstruction settings should be harmonized in a similar manner as proposed by the EANM guidelines for ^18^F-FDG tumor PET imaging^27^. In the present study, the PET/CT scans from young overweight/obese adults did not follow these guidelines, therefore we cannot guarantee that the recovery coefficients of the used reconstructions are the same. Moreover, methodological differences between cohorts did not allow us to check whether differences between HU and SUV thresholds are of different magnitude. Also the use of different cooling techniques (cooling vests *vs*. mattresses) and protocols might have introduced some bias. This study included only healthy male adults. The results should be applicable to other populations, such as women and men with different fat distributions, although this should be verified by replication in other cohorts with larger sample size. Moreover, biopsies of BAT-classical depots would be necessary to identify the density window (in terms of HU) of this tissue in different populations.

## Conclusions

BAT volume and activity as determined by ^18^F-FDG PET/CT highly depend on the quantification criteria used. Future human BAT studies should conduct sensitivity analysis with different thresholds in order to understand whether results are driven by the selected HU and SUV thresholds. According to our findings, when following an individualized cooling protocol, SUV_peak_ is the most consistent marker of maximal BAT activity across study cohorts independent of the HU and SUV threshold used, which may therefore facilitate comparisons across studies. The design of the present study precludes providing any conclusive threshold, but before more definitive thresholds for HU and SUV are available, we support the use of BARCIST 1.0 criteria to facilitate interpretation of BAT characteristics between research groups.

## Electronic supplementary material

Supplementary Information
