# Supplementary material for: The impact of using BARCIST 1.0 criteria on quantification of BAT volume and activity in three independent cohorts of adults
Source: Sci Rep. 2018 Jun 4;8:8567. doi: 10.1038/s41598-018-26878-4 (PMC5986766; doi:10.1038/s41598-018-26878-4)
Supplement: Supplementary file 1 — Supplementary Information [file 41598_2018_26878_MOESM1_ESM.pdf]

The impact of using BARCIST 1.0 criteria on quantification of BAT volume and activity in three independent cohorts of adults

Borja Martinez-Tellez, Kimberly J. Nahon, Guillermo Sanchez-Delgado, Gustavo Abreu-Vieira, Jose M. Llamas-Elvira, Floris HP. van Velden, Lenka Pereira Arias-Bouda, Patrick C.N. Rensen, Mariëtte R. Boon, Jonatan R. Ruiz

Table S1. Hounsfield Units and standardized uptake value thresholds and software used in BAT human studies from 1st of January 2007 to 10th of March 2017

Studies	Hounsfield units	Standardized uptake values	Software to quantify BAT
Hadi et al. 2007 [1]	NR	NR	NR
Kim et al. 2008 [2]	NR	NR	NR
Alkhawaldeh et al. 2008 [3]	NR	NR	NR
Basu et al. 2008 [4]	NR	NR	NR
Zukotynski et al. 2009 [5]	NR	NR	NR
Cypess et al. 2009 [6]	-250,-50	2.0	PET/CT viewer shareware
Van Marken et al. 2009 [7]	NR	NR	PMOD 2.85
Virtanen et al. 2009 [8]	NR	NR	NR
Saito et al. 2009 [9]	NR	NR	VOX-BASE
Au-Yong et al. 2009 [10]	Not used	Not used	Leonardo workstation
Paidisetty et al. 2009 [11]	NR	NR	NR
Lee et al. 2010 [12]	-250,-50	2.0	NR
Skarulis et al. 2010 [13]	NR	NR	MEDx image
Aukema et al. 2010[14]	NR	NR	Osirix DICOM viewer
Pfannenberg et al. 2010 [15]	-250,-50	2.0	NR
Park et al. 2010 [16]	NR	NR	NR
Zukotynski et al. 2010 [17]	NR	NR	NR
Garcia et al. 2010 [18]	NR	NR	NR
Rakheja et al. 2011[19]	NR	NR	NR
Ouellet et al. 2011 [20]	-100,-10	1.0	MIM software
Pace et al. 2011[21]	-250,-50	NR	Volumetrix
Orava et al. 2011 [22]	NR	NR	NR
Vijgen et al. 2011 [23]	NR	NR	NR
Lee et al. 2011 [24]	NR	2.0	NR
Lee et al. 2011 [25]	NR	2.0	NR
Jacene et al. 2011 [26]	NR	NR	NR
Huang et al. 2011 [27]	-250,-50	2.0	OsiriX 64-bit software
Yoneshiro et al. 2011 [28]	NR	NR	VOX-BASE workstation
Yilmaz et al. 2011 [29]	NR	NR	NR
Yoneshiro et al. 2012 [30]	NR	2.0	VOX-BASE workstation
Vrieze et al. 2012 [31]	-250,-50	2.0	Hybrid Viewer; HERMES
Muzik et al. 2012 [32]	-250,-50	2.0	NR
Vijgen et al. 2012 [33]	NR	NR	PMOD 2.85
Chalfant et al. 2012 [34]	NR	NR	SliceOmatic image software
Vosselman et al. 2012 [35]	-180,-10	1.5	PMOD 3.0
Cypess et al. 2012 [36]	-250,-10	2.0	PET/CT Viewer shareware
Ouellet et al. 2012 [37]	-100, -10	1.0	NR
Bredella et al. 2012 [38]	-250, -50	70%SUVmax	PET/CT Viewer shareware
Miao et al. 2012 [39]	-250, -50	NR	PET/CT Viewer shareware
Vogel et al. 2012 [40]	NR	NR	Osirix DICOM viewer
Schlögl et al. 2013 [41]	-250, -10	2.0	SPM8
Ahmadi et al. 2013 [42]	-87, -10	NR	NR
Banzo et al. 2013 [43]	NR	NR	NR
Carey et al. 2013 [44]	-180, -10	1.0	Extended BrillianceWorkstation
Ruth et al. 2013 [45]	-200,-10	2.0	Mathworks, Natick, MA
Lee et al. 2013 [46]	NR	2.0	IDL software

Pasanisi et al. 2013 [47]	-250, -50	NR	Volumetrix
Sugita et al. 2013 [48]	NR	2.0	VOX-BASE workstation
Van Rooijen et al. 2013 [49]	-150, -50	NR	PMOD
Yonsehiro et al. 2013 [50]	NR	2.0	VOX-BASE workstation
Yoneshiro et al. 2013 [51]	NR	2.0	VOX-BASE workstation
Yoneshiro et al. 2013 [52]	NR	2.0	VOX-BASE workstation
Orava et al. 2013 [53]	NR	NR	NR
Muzik et al. 2013 [54]	-250,-50	2.0	AMIDE software
Chen et al. 2013 [55]	NR	2.0	NR
Vosselman et al. 2013 [56]	-180, -10	1.5	PMOD 3.0
Perkins et al. 2013 [57]	-250, -50	No limit	Syngo MI workplace
Bredella et al. 2013 [58]	-250, -50	70%SUVmax	PET/CT Viewer shareware
van der Lans et al. 2013 [59]	-180, -10	1.5	PMOD 3.0
Zhang et al. 2013 [60]	-250, -50	2.0	PET/CT viewer software
Admiraal et al. 2013 [61]	-250, -50	2.0	Hermes Hybrid Viewer
Admiraal et al. 2013 [62]	-250, -50	2.0	Hermes Hybrid Viewer
Persichetti et al. 2013 [63]	-250, -50	2.0	MIM software
Vijgen et al. 2013 [64]	NR	NR	PMOD
Boon et al. 2014 [65]	Not used	2.0	Hermes Hybrid Viewer
Lee et al. 2014 [66]	-300,-10	2.0	NR
Lee et al. 2014 [67]	-300, -10	2.0	Software built with IDL
Jang et al. 2014 [68]	NR	1.5	syngo.via software
Chondronikola et al. 2014 [69]	-100, -10	1.0	NR
Schopman et al. 2014 [70]	-250,-50	2.0	Hybrid Viewer; HERMES
Blondin et al. 2014 [71]	-150,-30	1.5	NR
Bakker et al. 2014 [72]	Not used	2.0	Hermes Hybrid Viewer
Zhang et al. 2014 [73]	-250, -50	2.0	NR
Matsushita et al. 2014 [74]	Not used	2.0	VOX-BASE workstation
Vosselman et al. 2014 [75]	-180, -10	1.5	NR
Zhang et al. 2014 [76]	NR	NR	PET/CT Viewer shareware
Choi et al. 2014 [77]	-250,-50	2.0	Extended Brilliance Workspace
Bredella et al. 2014 [78]	-250, -50	70%SUVmax	PET/CT Viewer shareware
Orava et al. 2014 [79]	Not used	1.14	Vinci 2.54.0 software
Cao et al. 2014 [80]	NR	NR	NR
Hanssen et al. 2015 [81]	-180, -10	1.5	PMOD 3.0
Hanssen et al. 2015 [82]	-180, -10	1.5	PMOD 3.0
Hanssen et al. 2015 [83]	-180, -10	1.5	PMOD 3.0
Blondin et al. 2015 [84]	-150, -30	1.5	NR
Blondin et al. 2015 [85]	-150, -30	1.5	NR
Dinas et al. 2015 [86]	Not used	Not limit	NR
Vosselman et al. 2015 [87]	-180, -10	1.5	PMOD 3.0
Cypess et al. 2015 [88]	-250,-10	2.0	PET/CT Viewer shareware
Nirengi et al. 2015 [89]	-300, -10	2.0	VOX-BASE workstation
Carey et al. 2015 [90]	-180,-10	2.0	Extended BrillianceWorkstation
Wei et al. 2015 [91]	-150,-30	2.5	PET/CT viewer shareware
Butler et al. 2015 [92]	-250,-50	2.0	NR
Raiko et al. 2015 [93]	NR	NR	NR
Wang et al. 2015 [94]	-250,-50	2.0	Volume Viewer software
Puar et al. 2016 [95]	-180, -10	1.5	Inveon Research software
Hanssen et al. 2016 [96]	-180, -10	1.5	PMOD 3.0

Singhal et al. 2016 [97]	-250, -10	1.0 to 30	Fiji Software
Oxguven et al. 2016 [98]	-250,-50	2.0	Advantage Windows Workstation 4.5
Chondronikola et al. 2016 [99]	-190, -30	1.5	MIM software
Chondronikola et al. 2016 [100]	NR	NR	NR
Gifford et al. 2016 [101]	-200, -1	No limits	NR
Yoneshiro et al. 2016 [102]	Not used	2.0	NR
Ramage et al. 2016 [103]	-150, -30	2.0	PMOD 3.409
Bahler et al. 2016 [104]	NR	NR	NR
Bahler et al. 2016 [105]	-250,-50	2.0	Hermes Hybrid Viewer
Salem et al. 2016 [106]	-150, -5	2.0	NR
Bahler et al. 2016 [107]	-250,-50	2.0	Hermes Hybrid Viewer
Van der Lans et al. 2016 [108]	-180, -10	1.5	PMOD 3.0
Gatidis et al. 2016 [109]	NR	NR	NR
Nirengi et al. 2016 [110]	NR	NR	NR
Shao et al. 2016 [111]	-100, -10	1.0	TrueD system
Hibi et al. 2016 [112]	Not used	2.0	VOX-BASE workstation
Chen et al. 2016 [113]	-180, -10	1.5	NR
Lee et al. 2016 [114]	-300, -10	2.0	Software built with IDL
Becker et al. 2016 [115]	-250, -50	3.0	AW version 4.6, GE Healthcare
Takx et al. 2016 [116]	-250, -50	2.0	TrueD system
Shao et al. 2016 [117]	-250, -50	2.0	Syngo True D system
Muzik et al. 2016 [118]	-250, -50	2.0	AMIDE software
Blondin et al. 2016 [119]	-150, -30	1.5	NR
Blondin et al. 2017 [120]	NR	NR	NR
Gerngrob et al. 2017 [121]	-250, -50	2.0	"SYNGO" workstation (Siemens)
Hussein et al. 2017 [122]	-190,-30	2.0	NR
Yoneshiro et al. 2017 [123]	-300, -10	2.0	NR

NR: Not reported or cited in the study.

REFERENCES

1. Hadi, M., Chen, C. C., Whatley, M., Pacak, K. & Carrasquillo, J. a. Brown fat imaging with (18)F-6-fluorodopamine PET/CT, (18)F-FDG PET/CT, and (123)I-MIBG SPECT: a study of patients being evaluated for pheochromocytoma. *J. Nucl. Med.* **48,** 1077–83 (2007).

2. Kim, S., Krynyckyi, B. R., Machac, J. & Kim, C. K. Temporal relation between temperature change and FDG uptake in brown adipose tissue. *Eur. J. Nucl. Med. Mol. Imaging* **35,** 984–989 (2008).

3. Alkhawaldeh, K. & Alavi, A. Quantitative assessment of FDG uptake in brown fat using standardized uptake value and dual-time-point scanning. *Clin. Nucl. Med.* **33,** 663–7 (2008).

4. Basu, S. & Alavi, A. Optimizing interventions for preventing uptake in the brown adipose tissue in FDG-PET. *Eur. J. Nucl. Med. Mol. Imaging* **35,** 1421–3 (2008).

5. Zukotynski, K. A. *et al.* Constant ambient temperature of 24 degrees C significantly reduces FDG uptake by brown adipose tissue in children scanned during the winter. *Eur. J. Nucl. Med. Mol. Imaging* **36,** 602–6 (2009).

6. Cypess, A. M. *et al.* Identification and importance of brown adipose tissue in adult humans. *N. Engl. J. Med.* **360,** 1509–17 (2009).

7. van Marken Lichtenbelt, W. D. *et al.* Cold-activated brown adipose tissue in healthy men. *N. Engl. J. Med.* **360,** 1500–8 (2009).

8. Virtanen, K. A. *et al.* Functional brown adipose tissue in healthy adults. *N. Engl. J. Med.* **360,** 1518–1525 (2009).

9. Saito, M. *et al.* High incidence of metabolically active brown adipose tissue in healthy adult humans: effects of cold exposure and adiposity. *Diabetes* **58,** 1526–1531 (2009).

10. Au-yong, I. T. H., Thorn, N., Ganatra, R., Perkins, A. C. & Symonds, M. E. Brown Adipose Tissue and Seasonal Variation in Humans. *October* **58,** (2009).

11. Paidisetty, S. & Blodgett, T. M. Brown fat: atypical locations and appearances encountered in PET/CT. *AJR. Am. J. Roentgenol.* **193,** 359–66 (2009).

12. Lee, P. *et al.* A critical appraisal of the prevalence and metabolic significance of brown adipose tissue in adult humans. 601–606 (2010). doi:10.1152/ajpendo.00298.2010.

13. Skarulis, M. C. *et al.* Thyroid hormone induced brown adipose tissue and amelioration of diabetes in a patient with extreme insulin resistance. *J. Clin. Endocrinol. Metab.* **95,** 256–62 (2010).

14. Aukema, T. S., Vogel, W. V, Hoefnagel, C. A. & Valdés Olmos, R. a. Prevention of brown adipose tissue activation in 18F-FDG PET/CT of breast cancer patients receiving neoadjuvant systemic therapy. *J. Nucl. Med. Technol.* **38,** 24–7 (2010).

15. Pfannenberg, C. *et al.* Impact of age on the relationships of brown adipose tissue with sex and adiposity in humans. *Diabetes* **59,** 1789–1793 (2010).

16. Park, S. A. *et al.* Normal physiologic and benign foci with F-18 FDG avidity on PET/CT in patients with breast cancer. *Nucl. Med. Mol. Imaging (2010).* **44,** 282–289 (2010).

17. Zukotynski, K. A. *et al.* Seasonal variation in the effect of constant ambient temperature of 24°C in reducing FDG uptake by brown adipose tissue in children. *Eur. J. Nucl. Med. Mol. Imaging* **37,** 1854–1860 (2010).

18. Garcia, C. *et al.* Effective reduction of brown fat FDG uptake by controlling environmental temperature prior to PET scan: An expanded case series. *Mol. Imaging Biol.* **12,** 652–656 (2010).

19. Rakheja, R., Ciarallo, A., Alabed, Y. Z. & Hickeson, M. Intravenous administration of diazepam significantly reduces brown fat activity on 18F-FDG PET/CT. *Am. J. Nucl. Med. Mol. Imaging* **1,** 29–35 (2011).

20. Ouellet, V. *et al.* Outdoor temperature, age, sex, body mass index, and diabetic status determine the prevalence, mass, and glucose-uptake activity of 18F-FDG-detected BAT in humans. *J. Clin. Endocrinol. Metab.* **96,** 192–199 (2011).

21. Pace, L. *et al.* Determinants of physiologic 18F-FDG uptake in brown adipose tissue in sequential PET/CT examinations. *Mol. Imaging Biol.* **13,** 1029–1035 (2011).

22. Orava, J. *et al.* Different metabolic responses of human brown adipose tissue to activation by cold and insulin. *Cell Metab.* **14,** 272–279 (2011).

23. Vijgen, G. H. E. J. *et al.* Brown adipose tissue in morbidly obese subjects. *PLoS One* **6,** 2–7 (2011).

24. Lee, P. *et al.* High prevalence of brown adipose tissue in adult humans. *J. Clin. Endocrinol. Metab.* **96,** 2450–2455 (2011).

25. Lee, P., Swarbrick, M. M., Zhao, J. T. & Ho, K. K. Y. Inducible brown adipogenesis of supraclavicular fat in adult humans. *Endocrinology* **152,** 3597–3602 (2011).

26. Jacene, H. A., Cohade, C. C., Zhang, Z. & Wahl, R. L. The relationship between patients' serum glucose levels and metabolically active brown adipose tissue detected by PET/CT. *Mol. Imaging Biol.* **13,** 1278–83 (2011).

27. Huang, Y.-C. *et al.* The relationship between brown adipose tissue activity and neoplastic status: an (18)F-FDG PET/CT study in the tropics. *Lipids Health Dis.* **10,** 238 (2011).

28. Yoneshiro, T. *et al.* Brown Adipose Tissue, Whole-Body Energy Expenditure, and Thermogenesis in Healthy Adult Men. *Obesity* **19,** 13–16 (2011).

29. Yilmaz, Y. *et al.* Association between the presence of brown adipose tissue and non-alcoholic fatty liver disease in adult humans. *Aliment. Pharmacol. Ther.* **34,** 318–23 (2011).

30. Yoneshiro, T., Aita, S., Kawai, Y., Iwanaga, T. & Saito, M. Nonpungent capsaicin analogs (capsinoids) increase energy expenditure through the activation of brown adipose tissue in humans. *Am. J. Clin. Nutr.* **95,** 845–850 (2012).

31. Vrieze, A. *et al.* Fasting and postprandial activity of brown adipose tissue in healthy men. *J. Nucl. Med.* **53,** 1407–1410 (2012).

32. Muzik, O., Mangner, T. J. & Granneman, J. G. Assessment of oxidative metabolism in brown fat using PET imaging. *Front. Endocrinol. (Lausanne).* **3,** 15 (2012).

33. Vijgen, G. H. E. J. *et al.* Increase in brown adipose tissue activity after weight loss in morbidly obese subjects. *J. Clin. Endocrinol. Metab.* **97,** E1229-33 (2012).

34. Chalfant, J. S. *et al.* Inverse association between brown adipose tissue activation and white adipose tissue accumulation in successfully treated pediatric malignancy. *Am. J. Clin. Nutr.* **95,** 1144–1149 (2012).

35. Vosselman, M. J. *et al.* Systemic β-adrenergic stimulation of thermogenesis is not accompanied by brown adipose tissue activity in humans. *Diabetes* **61,** 3106–3113 (2012).

36. Cypess, A. M. *et al.* Cold but not sympathomimetics activates human brown adipose tissue in vivo. *Proc. Natl. Acad. Sci. U. S. A.* **109,** 10001–10005 (2012).

37. Ouellet, V. *et al.* Brown adipose tissue oxidative metabolism contributes to energy expenditure during acute cold exposure in humans. *J. Clin. Invest.* **122,** 545–552 (2012).

38. Bredella, M. A. *et al.* Young women with cold-activated brown adipose tissue have higher bone mineral density and lower Pref-1 than women without brown adipose tissue: A study in women with anorexia nervosa, women recovered from anorexia nervosa, and normal-weight women. *J. Clin. Endocrinol. Metab.* **97,** 584–590 (2012).

39. Miao, Q. *et al.* Stability in brain glucose metabolism following brown adipose tissue inactivation in chinese adults. *AJNR. Am. J. Neuroradiol.* **33,** 1464–9 (2012).

40. Vogel, W. V. *et al.* Intervention to Lower Anxiety of 18F-FDG PET/CT Patients by Use of Audiovisual Imagery During the Uptake Phase Before Imaging. *J. Nucl. Med. Technol.* **40,** 92–98 (2012).

41. Schlögl, M. *et al.* Overfeeding over 24 hours does not activate brown adipose tissue in humans. *J. Clin. Endocrinol. Metab.* **98,** 1956–1960 (2013).

42. Ahmadi, N. *et al.* Accurate detection of metabolically active ‘brown’ and ‘white’ adipose tissues with computed tomography. *Acad. Radiol.* **20,** 1443–1447 (2013).

43. Banzo, J. *et al.* Extensive hypermetabolic pattern of brown adipose tissue activation on 18F-FDG PET/CT in a patient diagnosed of catecholamine-secreting para-vesical paraganglioma. *Rev. Esp. Med. Nucl. Imagen Mol.* **32,** 397–399 (2013).

44. Carey, A. L. *et al.* Ephedrine activates brown adipose tissue in lean but not obese humans. *Diabetologia* **56,** 147–155 (2013).

45. Ruth, M. R., Wellman, T., Mercier, G., Szabo, T. & Apovian, C. M. An automated algorithm to identify and quantify brown adipose tissue in human 18F-FDG-PET/CT scans. *Obesity (Silver Spring).* **21,** 1554–60 (2013).

46. Lee, P. *et al.* Cold-activated brown adipose tissue is an independent predictor of higher bone mineral density in women. *Osteoporos. Int.* **24,** 1513–1518 (2013).

47. Pasanisi, F. *et al.* Evidence of brown fat activity in constitutional leanness. *J. Clin. Endocrinol. Metab.* **98,** 1214–1218 (2013).

48. Sugita, J. *et al.* Grains of paradise (Aframomum melegueta) extract activates brown adipose tissue and increases whole-body energy expenditure in men. *Br. J. Nutr.* 1–6 (2013). doi:10.1017/S0007114512005715

49. van Rooijen, B. D. *et al.* Imaging cold-activated brown adipose tissue using dynamic T2*-weighted magnetic resonance imaging and 2-deoxy-2-[18F]fluoro-D-glucose positron emission tomography. *Invest. Radiol.* **48,** 708–14 (2013).

50. Yoneshiro, T. *et al.* Recruited brown adipose tissue as an antiobesity agent in humans. *J. Clin. Invest.* **123,** 3404–3408 (2013).

51. Yoneshiro, T. *et al.* Impact of UCP1 and β3AR gene polymorphisms on age-related changes in brown adipose tissue and adiposity in humans. *Int. J. Obes. (Lond).* **37,** 993–998 (2013).

52. Yoneshiro, T. *et al.* Age-related decrease in cold-activated brown adipose tissue and accumulation of body fat in healthy humans. *Obesity (Silver Spring).* **19,** 1755–60 (2011).

53. Orava, J. *et al.* Blunted metabolic responses to cold and insulin stimulation in brown adipose tissue of obese humans. *Obesity* **21,** 2279–2287 (2013).

54. Muzik, O. *et al.* 15O PET measurement of blood flow and oxygen consumption in cold-activated human brown fat. *J. Nucl. Med.* **54,** 523–31 (2013).

55. Chen, K. Y. *et al.* Brown fat activation mediates cold-induced thermogenesis in adult humans in response to a mild decrease in ambient temperature. *J. Clin. Endocrinol. Metab.* **98,** 1218–1223 (2013).

56. Vosselman, M. J. *et al.* Brown adipose tissue activity after a high-calorie meal in humans. *Am. J. Clin. Nutr.* **98,** 57–64 (2013).

57. Perkins, A. C., Mshelia, D. S., Symonds, M. E. & Sathekge, M. Prevalence and pattern of brown adipose tissue distribution of 18F-FDG in patients undergoing PET-CT in a subtropical climatic zone. *Nucl. Med. Commun.* **34,** 168–74 (2013).

58. Bredella, M. A., Fazeli, P. K., Lecka-Czernik, B., Rosen, C. J. & Klibanski, A. IGFBP-2 is a negative predictor of cold-induced brown fat and bone mineral density in young non-obese women. *Bone* **53,** 336–339 (2013).

59. van der Lans, A. A. J. J. *et al.* Cold acclimation recruits human brown fat and increases nonshivering thermogenesis. *J. Clin. Invest.* **123,** 3395–3403 (2013).

60. Zhang, Q. *et al.* Differences in the metabolic status of healthy adults with and without active brown adipose tissue. *Wien. Klin. Wochenschr.* **125,** 687–695 (2013).

61. Admiraal, W. M. *et al.* Cold-induced activity of brown adipose tissue in young lean men of South-Asian and European origin. *Diabetologia* **56,** 2231–2237 (2013).

62. Admiraal, W. M. *et al.* Combining 123I-metaiodobenzylguanidine SPECT/CT and 18F-FDG PET/CT for the assessment of brown adipose tissue activity in humans during cold exposure. *J Nucl Med* **54,** 208–212 (2013).

63. Persichetti, A. *et al.* Prevalence, Mass, and Glucose-Uptake Activity of 18F-FDG-Detected Brown Adipose Tissue in Humans Living in a Temperate Zone of Italy. *PLoS One* **8,** 1–8 (2013).

64. Vijgen, G. H. E. J. *et al.* Vagus nerve stimulation increases energy expenditure: relation to brown adipose tissue activity. *PLoS One* **8,** e77221 (2013).

65. Boon, M. R. *et al.* Supraclavicular Skin Temperature as a Measure of 18F-FDG Uptake by BAT in Human Subjects. *PLoS One* **9,** e98822 (2014).

66. Lee, P. *et al.* Irisin and FGF21 are cold-induced endocrine activators of brown fat function in humans. *Cell Metab.* **19,** 302–309 (2014).

67. Lee, P. *et al.* Temperature-acclimated brown adipose tissue modulates insulin sensitivity in humans. *Diabetes* **177,** 1–59 (2014).

68. Jang, C. *et al.* Infrared thermography in the detection of brown adipose tissue in humans. *Physiol. Rep.* **2,** 1–7 (2014).

69. Chondronikola, M. *et al.* Brown Adipose Tissue Improves Whole Body Glucose Homeostasis and Insulin Sensitivity in Humans. *Diabetes* **63,** 4089–4099 (2014).

70. Schopman, J. E. *et al.* (18)F-fluorodeoxyglucose uptake in brown adipose tissue during insulin-induced hypoglycemia and mild cold exposure in non-diabetic adults. *Metabolism.* **63,** 1280–6 (2014).

71. Blondin, D. P. *et al.* Increased brown adipose tissue oxidative capacity in cold-acclimated humans. *J. Clin. Endocrinol. Metab.* **99,** 438–446 (2014).

72. Bakker, L. E. H. *et al.* Brown adipose tissue volume in healthy lean south Asian adults compared with white Caucasians: a prospective, case-controlled observational study. *lancet. Diabetes Endocrinol.* **2,** 210–217 (2014).

73. Rajpathak, S. N. *et al.* The role of insulin-like growth factor-I and its binding proteins in glucose homeostasis and type 2 diabetes. *Diabetes. Metab. Res. Rev.* **25,** 3–12 (2009).

74. Matsushita, M. *et al.* Impact of brown adipose tissue on body fatness and glucose metabolism in healthy humans. *Int. J. Obes.* **38,** 812–817 (2014).

75. Vosselman, M. J., Vijgen, G. H. E. J., Kingma, B. R. M., Brans, B. & van Marken Lichtenbelt, W. D. Frequent extreme cold exposure and brown fat and cold-induced thermogenesis: a study in a monozygotic twin. *PLoS One* **9,** e101653 (2014).

76. Zhang, Z. *et al.* The prevalence and predictors of active brown adipose tissue in Chinese adults. *Eur. J. Endocrinol.* **170,** 359–66 (2014).

77. Choi, H. Y. *et al.* Implication of circulating irisin levels with brown adipose tissue and sarcopenia in humans. *J. Clin. Endocrinol. Metab.* **99,** 2778–2785 (2014).

78. Bredella, M. A., Gill, C. M., Rosen, C. J., Klibanski, A. & Torriani, M. Positive effects of brown adipose tissue on femoral bone structure. *Bone* **58,** 55–8 (2014).

79. Orava, J. *et al.* Brown adipose tissue function is accompanied by cerebral activation in lean but not in obese humans. *J. Cereb. Blood Flow Metab.* **34,** 1018–23 (2014).

80. Cao, Q. *et al.* A pilot study of FDG PET/CT detects a link between brown adipose tissue and breast cancer. *BMC Cancer* **14,** 126 (2014).

81. Hanssen, M. J. W. *et al.* Glucose uptake in human brown adipose tissue is impaired upon fasting-induced insulin resistance. *Diabetologia* **58,** 586–595 (2015).

82. Hanssen, M. J. W. *et al.* Short-term cold acclimation improves insulin sensitivity in patients with type 2 diabetes mellitus. *Nat. Med.* **21,** 6–10 (2015).

83. Hanssen, M. J. W. *et al.* Serum FGF21 levels are associated with brown adipose tissue activity in humans. *Sci. Rep.* **5,** 10275 (2015).

84. Blondin, D. P. *et al.* Contributions of white and brown adipose tissues and skeletal muscles to acute cold-induced metabolic responses in healthy men. *J. Physiol.* **593,** 701–14 (2015).

85. Blondin, D. P. *et al.* Selective Impairment of Glucose but Not Fatty Acid or Oxidative Metabolism in Brown Adipose Tissue of Subjects With Type 2 Diabetes. *Diabetes* **64,** 2388–97 (2015).

86. Dinas, P. C. *et al.* Association between habitual physical activity and brown adipose tissue activity in individuals undergoing PET-CT scan. *Clin. Endocrinol. (Oxf).* 1–8 (2014). doi:10.1111/cen.12620

87. Vosselman, M. J. *et al.* Low brown adipose tissue activity in endurance trained compared to lean sedentary men. *Int. J. Obes. (Lond).* 1–7 (2015). doi:10.1038/ijo.2015.130

88. Cypess, A. M. *et al.* Activation of Human Brown Adipose Tissue by a β3-Adrenergic Receptor Agonist. *Cell Metab.* **21,** 33–38 (2015).

89. Nirengi, S., Yoneshiro, T., Sugie, H., Saito, M. & Hamaoka, T. Human brown adipose tissue assessed by simple, noninvasive near-Infrared time-resolved spectroscopy. *Obesity* **23,** 973–980 (2015).

90. Carey, A. L. *et al.* Chronic ephedrine administration decreases brown adipose tissue activity in a randomised controlled human trial: implications for obesity. *Diabetologia* **58,** 1045–1054 (2015).

91. Wei, H. *et al.* A clinical approach to brown adipose tissue in the para-aortic area of the human thorax. *PLoS One* **10,** 1–18 (2015).

92. Hew-Butler, T. *et al.* Plasma irisin in runners and nonrunners: no favorable metabolic associations in humans. *Physiol. Rep.* **3,** e12262–e12262 (2015).

93. Raiko, J. *et al.* Brown adipose tissue triglyceride content is associated with decreased insulin sensitivity, independently of age and obesity. *Diabetes. Obes. Metab.* **17,** 516–9 (2015).

94. Wang, Q. *et al.* Brown adipose tissue activation is inversely related to central obesity and metabolic parameters in adult human. *PLoS One* **10,** e0123795 (2015).

95. Puar, T. *et al.* Genotype-dependent brown adipose tissue activation in patients with pheochromocytoma and paraganglioma. *J. Clin. Endocrinol. Metab.* **101,** 224–232 (2016).

96. Hanssen, M. J. W. *et al.* Short-term Cold Acclimation Recruits Brown Adipose Tissue in Obese Humans. *Diabetes* **65,** 1179–89 (2016).

97. Singhal, V. *et al.* Effect of Chronic Athletic Activity on Brown Fat in Young Women. *PLoS One* **11,** e0156353 (2016).

98. Ozguven, S., Ones, T., Yilmaz, Y., Turoglu, H. T. & Imeryuz, N. The role of active brown adipose tissue in human metabolism. *Eur. J. Nucl. Med. Mol. Imaging* **43,** 355–61 (2016).

99. Chondronikola, M. *et al.* Brown Adipose Tissue Is Linked to a Distinct Thermoregulatory Response to Mild Cold in People. *Front. Physiol.* **7,** 129 (2016).

100. Chondronikola, M. *et al.* Brown Adipose Tissue Activation Is Linked to Distinct Systemic Effects on Lipid Metabolism in Humans. *Cell Metab.* **23,** 1200–6 (2016).

101. Gifford, A., Towse, T. F., Walker, R. C., Avison, M. J. & Welch, E. B. Characterizing Active and Inactive Brown Adipose Tissue in Adult Humans Using PET-CT and MR Imaging. *Am. J. Physiol. - Endocrinol. Metab.* ajpendo.00482.2015 (2016). doi:10.1152/ajpendo.00482.2015

102. Yoneshiro, T. *et al.* Brown adipose tissue is involved in the seasonal variation of cold-induced thermogenesis in humans. *Am. J. Physiol. Regul. Integr. Comp. Physiol.* ajpregu.00057.2015 (2016). doi:10.1152/ajpregu.00057.2015

103. Ramage, L. E. *et al.* Glucocorticoids Acutely Increase Brown Adipose Tissue Activity in Humans, Revealing Species-Specific Differences in UCP-1 Regulation. *Cell Metab.* **24,** 130–141 (2016).

104. Bahler, L. *et al.* Differences in Sympathetic Nervous Stimulation of Brown Adipose tissue between the young and old and the lean and obese. *J. Nucl. Med.* **57,** 1–27 (2015).

105. Bahler, L., Holleman, F., Booij, J., Hoekstra, J. B. & Verberne, H. J. Interobserver and intraobserver variability for the assessment of brown adipose tissue activity on 18F-FDG PET-CT. *Nucl. Med. Commun.* 1 (2015). doi:10.1097/MNM.0000000000000450

106. Salem, V. *et al.* Glucagon increases energy expenditure independently of brown adipose tissue activation in humans. *Diabetes, Obes. Metab.* **18,** 72–81 (2016).

107. Bahler, L., Deelen, J. W., Hoekstra, J. B., Holleman, F. & Verberne, H. J. Seasonal influence on stimulated BAT activity in prospective trials: a retrospective analysis of BAT visualized on 18F-FDG PET-CTs and 123I-mIBG SPECT-CTs. *J. Appl. Physiol.* **120,** 1418–23 (2016).

108. van der Lans, A. a. J. J., Vosselman, M. J., Hanssen, M. J. W., Brans, B. & van Marken Lichtenbelt, W. D. Supraclavicular skin temperature and BAT activity in lean healthy adults. *J. Physiol. Sci.* **66,** 77–83 (2016).

109. Gatidis, S. *et al.* Is It Possible to Detect Activated Brown Adipose Tissue in Humans Using Single-Time-Point Infrared Thermography under Thermoneutral Conditions? Impact of BMI and Subcutaneous Adipose Tissue Thickness. *PLoS One* **11,** e0151152 (2016).

110. Nirengi, S. *et al.* Assessment of human brown adipose tissue density during daily ingestion of thermogenic capsinoids using near-infrared time-resolved spectroscopy. *J. Biomed. Opt.* **21,** 91305 (2016).

111. Shao, X., Shao, X., Wang, X. & Wang, Y. Characterization of brown adipose tissue 18F-FDG uptake in PET/CT imaging and its influencing factors in the Chinese population. *Nucl. Med. Biol.* **43,** 7–11 (2016).

112. Hibi, M. *et al.* Brown adipose tissue is involved in diet-induced thermogenesis and whole-body fat utilization in healthy humans. *Int. J. Obes. (Lond).* **40,** 1655–1661 (2016).

113. Chen, Y. *et al.* Exosomal microRNA miR-92a concentration in serum reflects human brown fat activity. *Nat. Commun.* **7,** 11420 (2016).

114. Lee, P. *et al.* Brown Adipose Tissue Exhibits a Glucose-Responsive Thermogenic Biorhythm in Humans. *Cell Metab.* **23,** 1–8 (2016).

115. Becker, A. S., Nagel, H. W., Wolfrum, C. & Burger, I. A. Anatomical Grading for Metabolic Activity of Brown Adipose Tissue. *PLoS One* **11,** e0149458 (2016).

116. Takx, R. A. P. *et al.* Supraclavicular Brown Adipose Tissue 18F-FDG Uptake and Cardiovascular Disease. *J. Nucl. Med.* **57,** 1221–5 (2016).

117. Shao, X. *et al.* The role of active brown adipose tissue (aBAT) in lipid metabolism in healthy Chinese adults. *Lipids Health Dis.* **15,** 138 (2016).

118. Muzik, O., Mangner, T. J., Leonard, W. R., Kumar, A. & Granneman, J. G. Sympathetic Innervation of Cold-Activated Brown and White Fat in Lean Young Adults. *J. Nucl. Med.* (2016). doi:10.2967/jnumed.116.180992

119. Blondin, D. P. *et al.* Four-week cold acclimation in adult humans shifts uncoupling thermogenesis from skeletal muscles to brown adipose tissue. *J. Physiol.* (2016). doi:10.1113/JP273395

120. Blondin, D. P. *et al.* Inhibition of Intracellular Triglyceride Lipolysis Suppresses Cold-Induced Brown Adipose Tissue Metabolism and Increases Shivering in Humans. *Cell Metab.* **25,** 438–447 (2017).

121. Gerngroß, C., Schretter, J., Klingenspor, M., Schwaiger, M. & Fromme, T. Active brown fat during 18 FDG-PET/CT imaging defines a patient group with characteristic traits and an increased probability of brown fat redetection. *J. Nucl. Med.* jnumed.116.183988 (2017). doi:10.2967/jnumed.116.183988

122. Hussein, S. *et al.* Automatic Segmentation and Quantification of White and Brown Adipose Tissues from PET/CT Scans. *IEEE Trans. Med. Imaging* **36,** 734–744 (2017).

123. Yoneshiro, T. *et al.* Tea catechin and caffeine activate brown adipose tissue and increase cold-induced thermogenic capacity in humans. *Am. J. Clin. Nutr.* ajcn144972 (2017). doi:10.3945/ajcn.116.144972

Table S2. Lin´s concordance coefficient for the inter-observer reliability of BAT volume and activity by study cohort and by threshold of HU and SUV for quantification of BAT.

	BARCIST 1.0	HU: -180, -10; SUV: 1.5	HU: -250, -50; SUV: 2.0	HU: NA; SUV: 2.0
Young normal-weight adults				
Volume	0.962 (0.858 - 0.990)	0.953 (0.833 - 0.987)	0.976 (0.908 - 0.994)	0.951 (0.827 - 0.987)
SUV_{mean}	0.980 (0.930 - 0.934)	0.964 (0.877 - 0.990)	0.982 (0.933 - 0.996)	0.968 (0.908 - 0.989)
SUV_{peak}	1.000	1.000	1.000	1.000
Young overweight-obese adults				
Volume	0.990 (0.961 - 0.997)	0.993 (0.973 - 0.998)	0.993 (0.974 - 0.998)	0.986 (0.954 - 0.996)
SUV_{mean}	0.997 (0.989 - 0.999)	0.996 (0.985 - 0.999)	0.992 (0.975 - 0.998)	0.989 (0.961 - 0.997)
SUV_{peak}	0.996 (0.984 - 0.999)	0.996 (0.984 - 0.999)	0.996 (0.986 - 0.999)	0.996 (0.984 - 0.999)
Middle-aged overweight-obese adults				
Volume	0.996 (0.983 - 0.999)	0.992 (0.972 - 0.998)	0.999 (0.994 - 1.000)	0.983 (0.941 - 0.995)
SUV_{mean}	0.999 (0.998 - 1.000)	0.999 (0.998 - 1.000)	1.000 (0.999 - 1.000)	0.961 (0.951 - 0.997)
SUV_{peak}	0.983 (0.935 - 0.995)	0.982 (0.935 - 0.995)	0.993 (0.987 - 0.996)	0.906 (0.695 - 0.973)

Data are means and 95% confidence intervals.

Strength of the agreement: from 1.000 to 0.999 (almost perfect), from <0.999 to 0.95 (substantial), from <0.95 to 0.90 (moderate), <0.90 (poor).

BARCIST 1.0: HU:-190,-10; SUV: Individualized [1.2/(lean body mass/body mass)]; BAT: Brown adipose tissue; BMI: Body mass index; HU: Hounsfield units; SUV: Standardized uptake value.

Table S3. Absolute and relative (%) differences between thresholds in brown adipose tissue volume and activity by study cohort.

		Volume (ml)			SUV$_{mean}$ (g/ml)			SUV$_{peak}$ (g/ml)		
Young lean adults		Mean	95%CI		Mean	95%CI		Mean	95%CI	
BARCIST 1.0 vs. HU: NA; SUV: 2.0	Absolute	249***	186	312	0.4**	0.2	0.6	0	0	0.1
	%	+155			-9			-0.2		
BARCIST 1.0 vs. HU: -250, -50; SUV: 2.0	Absolute	92**	79	106	0.8***	0.6	0.9	0.1	0	0.2
	%	-57			+18			-0.8		
BARCIST 1.0 vs. HU: -180, -10; SUV: 1.5	Absolute	0	-5	5	0	0	0.1	0	0	0
	%	0			0			0		
HU: -250, -50; SUV: 2.0 vs. HU: NA; SUV: 2.0	Absolute	341***	267	415	1.2***	0.9	1.4	0.2	0	0.3
	%	+500			-23			+1		
HU: -250, -50; SUV: 2.0 vs. HU: -180, -10; SUV: 1.5	Absolute	92***	77	108	0.8***	0.6	1.0	0.1	0	0.2
	%	+135			-16			+1		
HU: -180, -10; SUV: 1.5vs. HU: NA; SUV: 2.0	Absolute	249***	186	311	0.4	0.1	0.6	0	0	0.1
	%	+155			-9			0		
Young overweight/obese adults										
BARCIST 1.0 vs. HU: NA; SUV: 2.0	Absolute	244**	114	374	0.6	0.1	1.1	0.1	0.3	0.1
	%	+207			-16			+1		
BARCIST 1.0 vs. HU: -250, -50; SUV: 2.0	Absolute	49**	29	70	0.1	0	0.3	0.6	0.2	1.1
	%	-42			+3			-6		
BARCIST 1.0 vs. HU: -180, -10; SUV: 1.5	Absolute	47**	24	69	0.5*	0.4	0.7	0	0	0
	%	+40			-14			0		
HU: -250, -50; SUV: 2.0 vs. HU: NA; SUV: 2.0	Absolute	294**	146	442	0.7*	0.3	1.2	0.7	0.2	1.2
	%	+430			-19			+7		
HU: -250, -50; SUV: 2.0 vs. HU: -180, -10; SUV: 1.5	Absolute	96***	64	128	0.7*	0.6	0.8	0.6	0.2	1.1
	%	+141			-17			+6		
HU: -180, -10; SUV: 1.5vs. HU: NA; SUV: 2.0	Absolute	198**	73	322	0.1	-0.3	0.4	0.1	0.1	0.3
	%	+120			-2			+1		
Middle-aged overweight/obese adults										

BARCIST 1.0 vs. HU: NA; SUV: 2.0	Absolute	106**	42	170	0.1	0.1	0.3	0.6*	0.2	0.9
	%	+124			+6			+10		
BARCIST 1.0 vs. HU: -250, -50; SUV: 2.0	Absolute	46**	28	64	0.3*	0.2	0.5	0.3	0.1	0.8
	%	-54			+13			-6		
BARCIST 1.0 vs. HU: -180, -10; SUV: 1.5	Absolute	38**	18	58	0.3*	0.2	0.4	0	0	0.1
	%	+45			-11			-0.7		
HU: -250, -50; SUV: 2.0 vs. HU: NA; SUV: 2.0	Absolute	152**	78	226	0.2	0	0.4	0.9*	0.3	1.5
	%	+384			-6			+16		
HU: -250, -50; SUV: 2.0 vs. HU: -180, -10; SUV: 1.5	Absolute	84***	60	108	0.6*	0.5	0.7	0.3	0.2	0.8
	%	+213			-21			+5		
HU: -180, -10; SUV: 1.5vs. HU: NA; SUV: 2.0	Absolute	68	13	123	0.4*	0.3	0.5	0.6*	0.3	0.9
	%	+55			+19			+10		

Data are means and 95% confidence intervals.

BMI: Body mass index; HU: Hounsfield units; BARCIST 1.0: HU:-190,-10; SUV: Individualized [1.2/(lean body mass/body mass)]; NA: Not applied; SUV: Standardized uptake value. * P≤0.05, ** P≤0.01, *** P≤0.001. See Figure 1 for graphical representation.

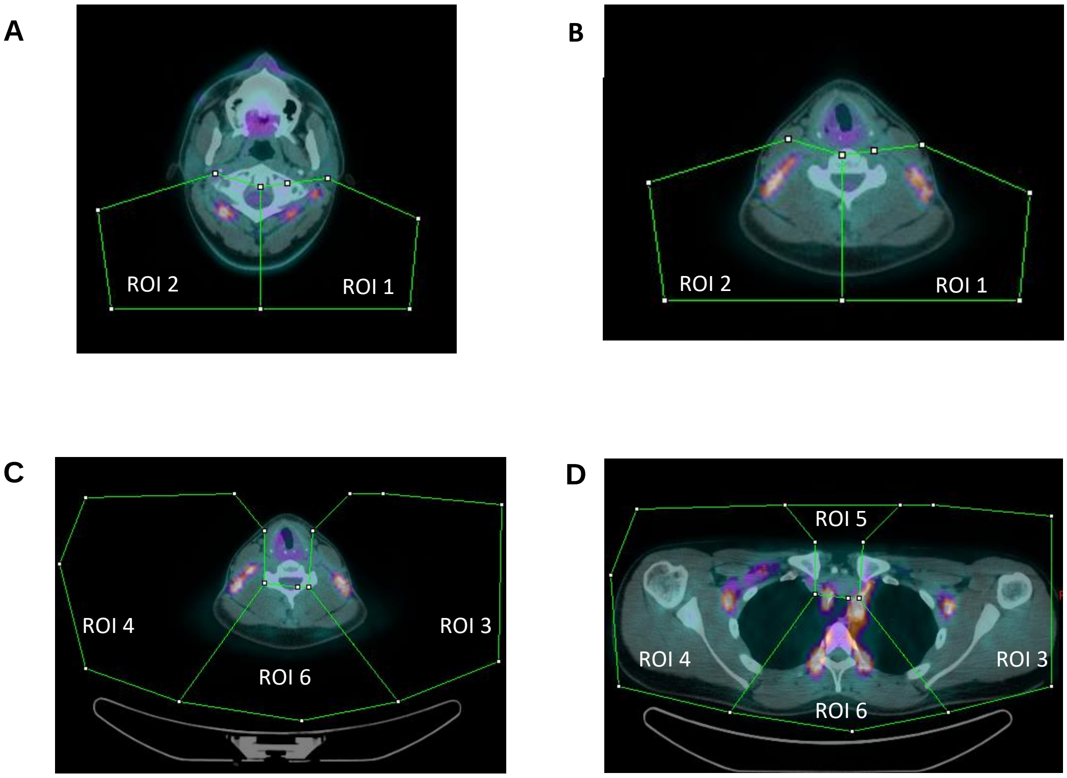

Figure S1. 3D-Axial technique: definition of the region of interest (ROI) drawn in the ^{18}F-fluorodeoxyglucose-Positron Emission Tomography/Computed Tomography images of a representative individual. **A.** ROI 1 and 2 in *atlas*. **B.** ROIs 1 and 2 in the end *cervical vertebrae 6.* **C.** ROI 3, 4 and 6 in the beginning of *cervical vertebrae 7*. **D.** ROI 3, 4, 5 and 6 in *thoracic vertebrae 4.*

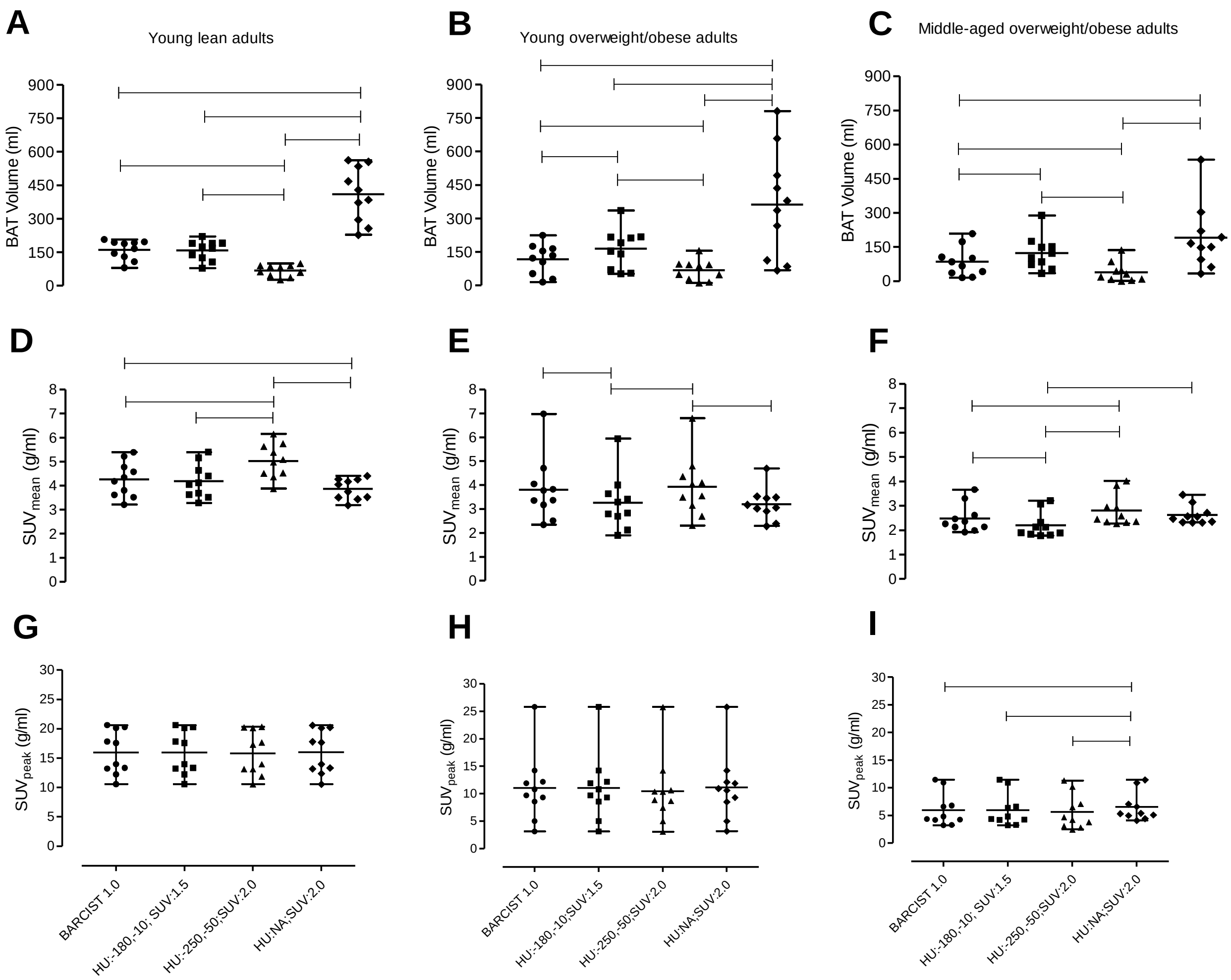

Figure S2. Brown adipose tissue (BAT) volume and activity determined by various thresholds of Hounsfield unit (HU) and Standardized uptake value (SUV) for three study cohorts. BAT volume (A-C), SUV_{mean} (D-F), and SUV_{peak} (G-I) were determined in young lean adults (A, D, G), young overweight/obese adults (B, E, H), and middle-aged overweight/obese adults (C, F, I). Data are means and 95% confident interval (n=10 per cohort). Significant differences between thresholds are indicated by parallel horizontal bars (all P≤0.05). BARCIST 1.0: HU:-190,-10; SUV: Individualized [1.2/(lean body mass/body mass)]; BMI: Body mass index; NA: Not applied. See Table S4 for exact absolute and relative differences between thresholds.
